# In pop pursuit: social bond strength predicts vocal synchrony during cooperative mate guarding in bottlenose dolphins

**DOI:** 10.1098/rstb.2023.0194

**Published:** 2024-06-23

**Authors:** Emma Chereskin, Simon J. Allen, Richard C. Connor, Michael Krützen, Stephanie L. King

**Affiliations:** ^1^ School of Biological Sciences, University of Bristol, Bristol BS8 1TQ, UK; ^2^ Evolutionary Genetics Group, Department of Evolutionary Anthropology, University of Zurich, Zurich CH-8057, Switzerland; ^3^ School of Biological Sciences, University of Western Australia, Crawley WA 6009, Australia; ^4^ Biology Department, University of Massachusetts Dartmouth, North Dartmouth, MA 02747, USA; ^5^ Institute of Environment and Department of Biological Sciences, Florida International University, North Miami, FL 33181, USA

**Keywords:** bottlenose dolphin, cooperation, mate guarding, social networks, vocal synchrony, alliances

## Abstract

Vocal communication is an emblematic feature of group-living animals, used to share information and strengthen social bonds. Vocalizations are also used to coordinate group-level behaviours in many taxa, but little is known of the factors that may influence vocal behaviour during cooperative acts. Allied male Indo-Pacific bottlenose dolphins (*Tursiops aduncus*) use the ‘pop’ vocalization as a coercive signal when working together to herd single oestrous females. Using long-term association and acoustic data, we examined the influence of social and non-social factors on pop use by allied male dolphins in this context. Neither pop rate nor pop bout duration were influenced by any of the factors examined. However, allied males with stronger social bonds engaged in higher rates of vocal synchrony; whereby they actively matched the timing of their pop production. Hence, social bond strength influenced pop use in a cooperative context, suggesting dual functions of pop use: to induce the female to remain close, and to promote social bond maintenance and cooperation among males.

This article is part of the theme issue ‘The power of sound: unravelling how acoustic communication shapes group dynamics’.

## 1. Introduction

Individuals commonly use vocalizations to navigate their social landscape, with vocal communication an emblematic feature of many group-living animals. Vocalizations are an efficient signalling method due to their long-range capacity, low energetic cost and flexibility [1–3]. Furthermore, they can be used for a variety of functions, such as alarm calling [4–6], advertising individual identity or quality [7–10], mediating social interactions [11–14] and coordinating group behaviours [15–17].

Indeed, vocalizations are used to coordinate group-level behaviours in many taxa, such as African wild dogs [17], meerkats [18], red-fronted lemurs [19] and jackdaws [15], wherein vocal signals are used to reach consensus and initiate group movement or other behaviours. In some species, vocalizations are used not only to coordinate group-level behaviours, but also to cooperate in achieving joint action tasks. The current body of evidence suggests that, outside of humans, both chimpanzees and bottlenose dolphins use communication signals to actively coordinate collaboration [20–24]. For example, chimpanzees in human care use communicative gestures to ensure successful cooperation to gain a food reward [21]. In wild chimpanzees, bark vocalizations are associated with increased hunting party recruitment and hunting success [24]. Bottlenose dolphins in human care have also been shown to use vocalizations to facilitate the coordination of a cooperative button-pressing task [20,23].

Vocal signal production can be influenced by a variety of external factors. The social environment, for instance, can influence patterns of vocal communication, both in call type and intended receiver. Spider monkeys, for example, exchange more contact calls with, and match the specific call type of, those group members with whom they share greater affiliative bonds [25]. Ring-tailed lemurs exhibit a vocal partner preference driven by their social bonds, reserving calls for their closest affiliates [11]. Male bottlenose dolphins exchange identity whistles with their alliance members, but do so preferentially with those with whom they share weaker bonds and less affiliative physical contact, serving to maintain these weaker yet still crucial alliance relationships [26]. Non-social factors can also influence an individual’s vocal behaviour. Birdsong has been shown to vary according to environmental cues, such as elevation and tree cover [27]. Furthermore, anthropogenic noise can cause some species to alter their vocal behaviour by increasing amplitude, duration or changing call type, for example, to compensate for the effect of noise masking [20,28]. However, there has been little investigation into the external factors that may drive variation in vocal production patterns during group cooperative events. Here, we examine both social and non-social external factors that may influence the production of a vocalization used in a polyadic, cooperative context in wild bottlenose dolphins.

In Shark Bay, Western Australia, Indo-Pacific bottlenose dolphins (*Tursiops aduncus*) display highly differentiated social relationships in the context of a fission–fusion grouping pattern in an open social network, wherein social group composition changes over time and behavioural contexts [29,30]. Unrelated adult male dolphins in this population form decades-long, multi-level alliances at three levels, the core social unit of which is the second-order alliance consisting of 4–14 individuals [30]. From within the second-order alliance, males will recruit first-order allies to work cooperatively in pairs or trios to herd and guard single oestrous females in events termed ‘consortships’ [31,32]. The stability of first-order alliances can be highly variable, with some males having consistent first-order allies and others consorting females with numerous males from within their second-order alliance in a given mating season [30,33–35]. Consortships last from hours to weeks, during which time these first-order allies must work together to keep the female with them and guard against attempted thefts by rival alliances [30,32]. During consortships, males can display aggression towards the female, both physically and through vocalizations termed ‘pops’, which compel the female to remain close to the males [36,37]. Pops are produced only by males, with adult females having never been observed or recorded producing pops in over four decades of research [36–39]. Pops are narrow-band, low frequency, pulsed calls that are produced in repetitive sequences (called ‘trains’), and are almost exclusively used by males during consortships [36,37]. Pop trains can vary in both the number of individual pops they contain and in the pop rate (pops per second), ranging from 3 to 30 pops per train and 6–12 pops per second [36,38]. Allied males will also coordinate pop production by actively matching each other’s tempo and synchronizing their pop trains [38]. As with motor synchrony in this population, the use of vocal synchrony is thought to express social bonds and promote cooperative behaviour [38,40–42]. Pop production also significantly increases when the closest male to the female changes, termed ‘guard switching’, illustrating the importance of this vocalization when coordinating behaviour during a cooperative event [39].

It remains unknown whether other social and non-social factors can influence the use of this vocalization in a cooperative context. First, as pops are used to coerce females and induce them to remain close [36,37,39], we expect that the distance a female is being herded from outside of her preferred home range will be a strong non-social predictor of pop production [43]. However, given that males must work together to herd the female, the strength of their social bonds and their history of consorting together may be social factors that directly influence their vocal behaviour. Here, we examine the influence of first-order alliance fidelity, male cumulative social bond strength, and distance from the consorted female’s home range, on the variation in pop production during consortships.

## 2. Methods

Data for this study were collected in the eastern gulf of Shark Bay, Western Australia, where our long-term research on a free-ranging population of Indo-Pacific bottlenose dolphins has been ongoing since 1982. We used data from 22 focal males from five second-order alliances: RR (3 of 3 males); KS (6 of 8 males); PD (4 of 5 males); EC (3 of 8 males); and HG (6 of 9 males). All first-order alliances included in the analyses consisted of trios to ensure consistency in first-order alliance group size. Association data are collected annually during boat-based surveys, where a ‘survey’ is defined as a minimum 5 min observation during which predominant behaviour and group composition (as defined by the 10 m chain rule, where each dolphin in the group is within 10 m of any other dolphin [44]) are recorded. Individual dolphins are first identified in the field by experienced observers and confirmed using photographs of their dorsal fins, identified by distinct shapes, scars and notches [45].

### (a) Acoustic data collection

Acoustic data were collected during follows of first-order alliances and their female consorts between 2016 and 2022, as per King *et al*. [34,39,41]. We towed four HTI-96 MIN series hydrophones (High Tech, Inc., Long Beach, MS; flat frequency response: 0.002–30 kHz ± 1 dB) at 1 m depth in a rectangular formation (*ca* 2.3 m × 3.5 m) to allow for localization of vocal signals to first-order alliances. Recordings were made onto a TASCAM DR-680 MKII multi-track recorder (TASCAM; TEAC Corp., Santa Fe Springs, CA) at a sampling rate of 96 kHz. A spoken track was used to note the location of the animals in relation to the boat using a compass bearing (wherein the boat’s bow is 0°), distance from the boat (m), group composition and group behaviour. Localized bearings were calculated from the centre of the array. Custom-written MATLAB (MathWorks, Natick, MA) routines were used to determine the localization error of the array by calculating two-dimensional averaged minimum number of receiver array (MINNA) localizations [46–48]. The array was calibrated using two different pop trains recorded from this population. The acoustic localization accuracy for pop bearings (*n* = 50 pops) was calculated as 100% within ±15° of the true location, 94% within ±10° and 68% within ±5° [38,39]. Variation in estimated direction within a train was low, with less than 2° difference between sequential pops in a train.

### (b) Social factors

Association data, based on photo-identification, collected during surveys between 1994 and 2022 were used to calculate association indices (see below) between all second-order alliance members and between first-order allies and the consorted females. The minimum number of sightings used to calculate association indices was 15 [34,49]. To ensure measures of association were comparable across surveys, only association data recorded within the first 5 min of the survey were used. Furthermore, ‘resights,’ wherein the same group was encountered within 2 h of the initial sighting, were excluded from the analysis to ensure the independence of observations. Finally, all surveys in which foraging was the predominant behavioural activity were excluded, as individuals can be attracted to the same foraging patch but are not necessarily associating preferentially [33].

Association indices were calculated as the simple ratio index (SRI) [50] in the R package *asnipe* [51], which is a measure of the proportion of time two animals spend together, with ‘0’ indicating a pair (or dyad) had never been observed together and ‘1’ indicating a pair is always seen together. Dyadic SRIs were calculated for all members of a second-order alliance. For each acoustic recording of a consortship, survey data for the allied males collected from the year the focal alliance had formed until the date of that acoustic recording were used to calculate association indices between the males. Second-order alliances form when males are in their early to mid-teens, and the males in this study formed their alliances at various times between 1994 and 2011 [52].

Social networks were then constructed using association indices, wherein nodes represent individuals and the edges that connect them represent relationship strengths. The R package *sna* [53] was used to calculate the node metric ‘strength’ for each male within their second-order alliance. Strength is defined as the sum of the edge weights (SRIs, see above) that are connected to each node and describes an individual’s connectedness within their social network, also referred to as their cumulative social bond strength [54–57]. As second-order alliance size varies, these individual strength values are normalized by dividing each value by the maximum strength value within the second-order alliance, scaling results between 0 and 1 [26,33,57,58]. At the first-order alliance level, these normalized strength values were then averaged across the three males to produce a mean strength value for each first-order alliance.

Fidelity to the first-order alliance was also calculated, defined as the proportion of total consortships that an individual male engaged in with a specific first-order alliance, with values ranging from 0 to 1, with ‘0’ meaning an individual had never consorted with that first-order alliance and ‘1’ meaning all consortships were performed with the same first-order alliance [30,35]. At the first-order alliance level, this value was averaged across the three males.

### (c) Non-social factor

The consorted female’s home range was calculated using GPS locations based on 95% kernel density estimates with a custom smoothing factor, as per Wild *et al*. [59], using the R package *adehabitatHR* [60]. GPS locations were taken during surveys collected both within and outside the mating season. The centroid of the home range was then calculated and the distance in km between the GPS location of the recording and that of the female’s home range centroid was then estimated, with the minimum number of sightings required to calculate home ranges being five.

### (d) Acoustic analysis

Spectrograms (fast Fourier transform (FFT): 1024, Blackman-Harris window) were examined visually using Adobe Audition v.13.0.13 (Adobe Inc., San Jose, CA) for instances of pop trains. Pops were grouped into trains that were separated by an inter-train interval (ITI), wherein the ITI was at least twice the length of the preceding inter-pop interval, and typically featured a short tonal component at approximately 5 kHz [38]. The number of pops for each train was tallied and any instances of multiple males popping together, i.e. producing synchronous pops, were also noted. The two are differentiated based on temporal patterning, with synchronous pop trains having shorter inter-pop intervals than single male pop trains, as per Moore *et al*. [38]. We used a MATLAB-based program (TOADy [48]) to localize pop trains to the consorting group. To allocate pop trains to a specific first-order alliance with confidence, pops had to be localized to a trio of males separated by greater than ±15° from conspecifics.

We then calculated several pop metrics to describe the vocal behaviour of the males in the first-order alliance. Pop rate was calculated as the number of pops in each train divided by the pop train duration, yielding a rate of pops per second. This was calculated for all pop trains that were not synchronous. Pop trains were then grouped into bouts, where a bout was defined as a sequence of pop trains with inter-train intervals of less than 2 s. Bout duration was calculated from the start of the first pop train to the end of the last pop train in the bout. We then calculated the proportion of pop trains produced during the recording that were synchronous by dividing the number of synchronous pop trains by the total number of pop trains produced by that trio.

### (e) Statistical analysis

To examine how first-order alliance fidelity, male cumulative social bond strength, and distance from the consorted female’s home range affected pop production at the first-order alliance level, we built several mixed-effect models using the *lme4* [61] package in R (electronic supplementary material, table S1). The dependent variables for each model were as follows: (a) pop rate (linear); (b) bout duration (linear); and (c) proportion of synchronous pops (generalized, binomial). For each model, the independent variables were as follows: (i) fidelity to the first-order alliance; (ii) average male cumulative social bond strength; and (iii) distance from the female’s home range centroid. We included first- and second-order alliance membership as random effects, and the amount of recording time per first-order alliance was included as an offset for models (a) and (b) and the total number of pop trains was included as an offset for model (c). For all models, model fit was assessed using the R package *DHARMa* [62] by visualizing model residuals, and the variance inflation factor (VIF) was calculated for the independent variables to test for collinearity using the *car* [63] package in R. VIF values were less than 3 for all models and, thus, all independent variables were retained [64]. All models were then standardized and ranked using the dredge function, and model averaging was performed on those with a delta Akaike information criterion (AIC) less than 2 using the *MuMIn* [65] package in R (electronic supplementary material, table S2). The R packages *effects* [63] and *ggplot2* [66] were used to plot the model estimates over the raw data.

## 3. Results

**Figure 1.**
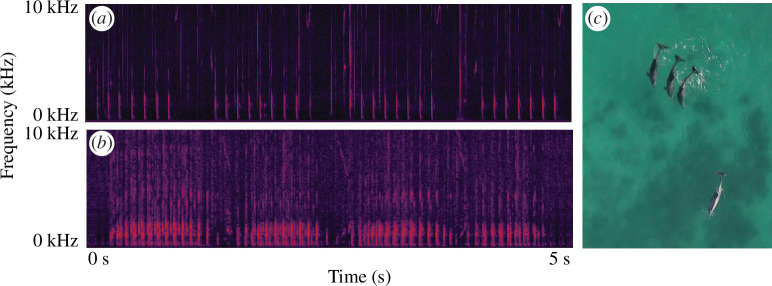
Vocalizations produced during a cooperative act in Indo-Pacific bottlenose dolphins. Spectrograms of pop train sequences from a single male (*a*) and multiple males (*b*) (sampled at 96 kHz, FFT length: 1024, Blackman–Harris window function). Typical herding behaviour (*c*), with three allied males (RR alliance, top centre) moving synchronously behind a female dolphin (bottom right) during a consortship in Shark Bay.

We analysed approximately 30 h of acoustic data (figure 1*a,b*) from a total of 14 unique consortship events (figure 1*c*), with 801 pop trains localized to 12 unique first-order alliances, across five second-order alliances (electronic supplementary material, table S3). There were no first-order alliance and female repeats, but two first-order alliances were sampled twice on different occasions (i.e. with different females).

### (a) No influence of social or geographical factors on pop rate or bout duration

Contrary to our predictions, at the first-order alliance level, we did not detect a significant effect of the distance from the consorted female's home range on pop rate (table 1*a*). Furthermore, we found no effect of alliance fidelity nor average male cumulative social bond strength on the pop rate. There was also no relationship between pop bout duration and alliance fidelity, male cumulative social bond strength, or distance from the female’s home range (table 1*b*).

**Table 1.** Results from mixed-effect models investigating social and non-social factors influencing pop production. We tested the effects of average fidelity to the first-order alliance, male cumulative social bond strength and distance from the female’s home range centroid on (*a*) average pop rate (*b*) bout duration and (*c*) proportion of synchronous pop trains for first-order alliances. Parameter estimates were averaged over the top model set based on AIC selection (ΔAIC ≤ 2). Model averaging was performed across three top models for (*a*) and (*c*). The top model set for (*b*) contained only one model therefore no averaging was conducted. We report the effect, standard error (s.e.) and confidence intervals (CI). Bold values indicate significant results (i.e. confidence intervals that do not intersect zero).

	effect	s.e.	CI (2.5%)	CI (97.5%)
**(*a*) pop rate**				
average alliance fidelity	−1.53	1.04	−3.95	0.89
average male cumulative social bond strength	1.28	1.11	−1.18	3.75
distance from female home range	1.24	1.09	−1.15	3.64
**(*b*) bout duration**				
average alliance fidelity	−4.09	6.51	−16.85	8.67
average male cumulative social bond strength	−3.35	6.16	−15.43	8.72
distance from female home range	−1.59	6.60	−14.53	11.34
**(*c*) synchronous pop trains**				
average alliance fidelity	0.15	0.54	−1.03	1.32
average male cumulative social bond strength	**2.55**	**1.01**	**0.27**	**4.82**
distance from female home range	−0.13	0.50	−1.22	0.95

### (b) Social bond strength predicts pop synchrony

**Figure 2.**
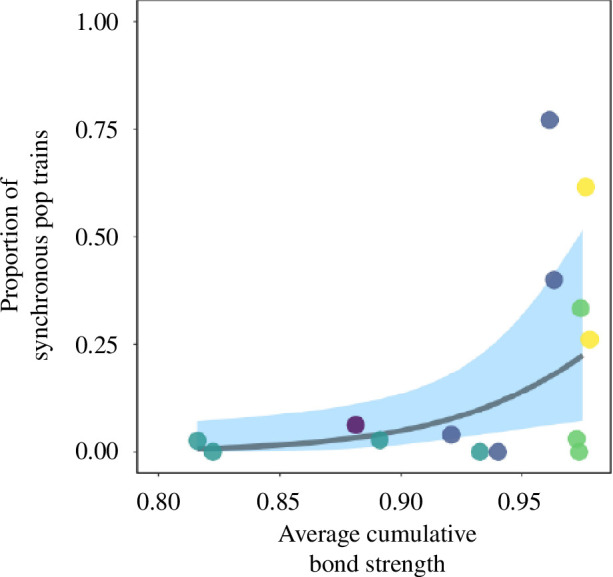
Influence of bond strength on synchronous pop production. The proportion of synchronized pops was calculated by dividing the number of synchronous pop trains by the total number of pop trains. The cumulative bond strength was calculated by summing an individual’s dyadic bond strengths within the second-order alliance, normalizing them, and then averaging those values across the first-order alliance. Panel shows raw data with model estimates (solid line) and 95% confidence intervals (shaded area). Data points are colour-coded by second-order alliance membership (RR, yellow; PD, green; EC, purple; HG, blue; KS, turquoise). First-order alliances that were more central within their second-order alliance displayed a higher proportion of synchronized pops.

First-order alliances that were on average more central within their second-order alliance, i.e. had greater cumulative social bond strength with their second-order allies, produced a significantly higher proportion of synchronous pop trains (figure 2, table 1*b*). We found no effect of alliance fidelity or distance from the female’s home range on the proportion of synchronized pop trains produced.

## 4. Discussion

Building on prior research [36–39], we examined the influence of social and non-social factors on the use of the pop vocalization by allied male dolphins during a cooperative event: the herding of oestrus females. We found that first-order alliances that were more central within their second-order alliance (i.e. had higher cumulative social bond strengths) engaged in higher rates of synchronous pop production. However, we did not detect any effect of first-order alliance fidelity, male cumulative bond strength or distance from the female’s home range on pop rate or bout duration.

First-order alliances that were more central within their second-order alliance displayed a higher proportion of synchronized pops compared with those that were less central. The relationships within a second-order alliance are highly differentiated, with males typically selecting first-order allies from those with whom they share a stronger social bond [33]. Furthermore, those males that are more central within their second-order alliance display higher consortship rates and consortship durations [33] and, ultimately, higher reproductive success [57]. Here, we suggest that more central males, and therefore those more successful at securing mating opportunities, use vocal synchrony at higher rates to further facilitate cooperation and reduce tension during consortship events (see also Connor *et al*. [40]). These results add to evidence suggesting that vocal synchrony, like other forms of behavioural synchrony, promotes cooperation and bonding between males during cooperative herding events, circumstances in which they are working together to secure a resource (paternities) that they cannot share [38,39].

Evidence for the use of synchrony in a cooperative alliance context is currently limited to dolphins and humans [40,67,68]. In humans, the use of physical synchrony can promote the release of oxytocin and aid in reinforcing social bonds [69–72]. Recent evidence demonstrates this phenomenon extends to vocalizations, with synchronous vocal behaviour promoting affiliation, enhanced memory and increased group coordination in humans [73]. The apparent convergence between humans and dolphins in the use of synchrony among allies in a cooperative context raises interesting questions regarding the evolution of collaboration and the tools used to facilitate it.

In addition to signalling cooperation among males, pop synchrony may function as a female-directed signal. First, pop synchrony may impress the female by advertising allied male quality. However, pop vocalizations are often associated with physically aggressive behaviour towards the female consort, with 36% of charges at the female and 69% of head jerks (both aggressive acts) occurring within 1 s of pop production [36], arguing against pops playing a functional role in impressing the female. Second, herding is a cooperative task and, by signalling a higher level of cooperation between the males herding her, pop synchrony may discourage female resistance. While females typically behave passively towards male consorts, they sometimes attempt to escape by swimming rapidly away, termed ‘bolting’. Male cooperation extends to attempting to recapture bolting females, not necessarily chasing directly behind the female but, instead, at angles to cut off her escape routes [32]. Thus, the higher level of cooperation signalled by synchronous pops may dissuade a female from such escape attempts. Third, vocal synchrony increases the number of pops a female consort is exposed to in a given train compared with solo pop trains. It is possible that females may interpret synchronous pops by males as a high-level threat vocalization, signalling the males’ intent to engage in physical aggression should the female not remain close [36,37]. Black-capped chickadees [74], tree shrews [75] and big brown bats [76], for example, all use elevated vocalization rates during increasingly agonistic interactions. ‘Bolting’ was not observed during the consortships analysed in this study and therefore could not be included as a predictor variable in our models. However, if the sole function of pop synchrony was to intimidate the female, we would not expect male cumulative social bond strength to be a significant predictor of synchronous pop production. Instead, it appears that synchronous pop production by males plays a role in both promoting cooperation among strongly bonded allies as well as signalling to females.

Contrary to our predictions based on the earlier hypothesis by Connor & Vollmer [43], we found no effect of distance from the female’s home range on male vocal behaviour. It may be worth revisiting this with a larger sample size. Further, while our study examined the effect of distance calculated from the centroid of the female home range, future research might explore the effect of home range overlap between the female’s home range and that of other male alliances. Connor & Vollmer [43] additionally suggested that, if a female’s home range overlaps with that of two or more alliances, the alliance in possession of the female may use elevated pop rates to deter the female escaping to the area of her home range where another alliance may more easily capture her [43]. It is possible that the cost of retrieving a female returning to a home range with a higher degree of overlap with rival alliances is greater than that of retrieving a female returning to a home range with lower overlap. As such, males may display increased aggressive intent via elevated pop rates to constrain the movement of their female consort when female home range overlap with rival alliances is higher. Therefore, although we found no effect of distance from the female’s home range centroid on pop production, the effect of home range overlap and habitat type on pop production still warrants further exploration.

While our sample size was relatively small, our findings demonstrate that male bottlenose dolphins vary their use of a vocal signal during a cooperative event: synchronizing pop trains more frequently among more central first-order allies. Social bond strength thus predicts the use of vocal synchrony in a cooperative mate-guarding context. In conclusion, we suggest that pops may serve a dual function: to induce the female to remain close, as well as to promote bonding and cooperation among the males.

## Data Availability

Code and data are provided in the electronic supplementary material [77].
